# Facile Phase Control and Photocatalytic Performance of BiVO_4_ Crystals for Methylene Blue Degradation

**DOI:** 10.3390/ijerph20043093

**Published:** 2023-02-10

**Authors:** Heshan Cai, Linmei Cheng, Huacong Chen, Rongni Dou, Junfeng Chen, Yuxin Zhao, Fuhua Li, Zheng Fang

**Affiliations:** 1 Biochar Engineering Technology Research Center of Guangdong Province, School of Environmental and Chemical Engineering, Foshan University, Foshan 528000, China; 2 School of Environmental Science and Engineering, Guangdong University of Petrochemical Technology, Maoming 525000, China; 3 School of Life Science, Qufu Normal University, Qufu 273165, China

**Keywords:** hydrothermal method, photocatalysis, bismuth vanadate, emerging contaminants, crystal structure

## Abstract

Emerging contaminants, which mainly exist as organic pollutants and pose adverse biological effects, could be removed using photocatalytic degradation, resulting in a low-cost and environmentally friendly solution. Herein, BiVO_4_ nanoparticles with different morphologies and photocatalytic performances were synthesized by hydrothermal treatment at different residence times. The XRD and SEM results indicate that the crystal phase of BiVO_4_ gradually transformed from a single tetragonal phase to a single monoclinic crystal phase as the hydrothermal time increased, and with the extension of the hydrothermal time, the morphology of BiVO_4_ nanoparticles gradually differentiated from a smooth spherical shape to flower-like shapes composed of polyhedrons; the size of the crystals also increased accordingly. Methylene blue (MB), used as a probe of organic pollutants, was degraded under visible light irradiation by all BiVO_4_ samples to investigate its photocatalytic activities. The experimental results show that the longer the hydrothermal time, the better the photocatalytic performance. The optimum hydrothermal time was 24 h, at which the sample showed the highest photocatalytic activity for MB degradation. This work shows a convenient strategy for control of the crystal phase of BiVO_4_-based photocatalysts based on the understanding of the crystal morphology evolution mechanism, which will benefit the researchers in designing new BiVO_4_-based photocatalysts with high efficiency for emerging contaminants’ degradation.

## 1. Introduction

The awareness of green eco-environmental protection has been enhanced due to the recent developments in social science and technology [1]. Photocatalytic technology has become increasingly important as a green and sustainable technology [2,3,4]. Emerging contaminants, such as microplastics, persistent organic pollutants, antibiotics, pesticides, and endocrine disruptor chemicals, could damage the reproduction of human beings and animals [5,6,7]. Most of the emerging contaminants exist as organic pollutants and could be removed by the adsorptive and photocatalytic approach, which has recently gained broad attention [8,9,10].

BiVO_4_ is a new type of visible light catalyst that has become popular due to its nontoxicity, high photostability, low cost of production, narrow band gap, response bands in visible light regions, and other advantages [3,11]. The crystallinity phase of BiVO_4_ can be easily controlled by adjusting preparation conditions, playing a vital role in its photocatalytic efficiency, which is a low-cost strategy for enhancing the photocatalytic performance for water treatment [12]. It has been found that there are three main crystal structures in the BiVO_4_: a tetrahedral scheelite structure; monoclinic scheelite structure; and tetrahedral zircon structure [13]. Among these, the band gap (*E*g) of the BiVO_4_ with monoclinic and tetrahedral scheelite structures is approximately 2.4 eV [14], and that of the tetrahedral zircon structure is approximately 2.9 eV. Although monoclinic BiVO_4_ and tetrahedral BiVO_4_ have similar crystal structures [15], the BiVO_4_ with monoclinic scheelite structures has good visible light catalytic properties. At the same time, the BiVO_4_ with tetrahedral zircon structures has poor photocatalytic performances, indicating that the crystal structures have major influences on the photocatalytic properties of the BiVO_4_. The results of this study showed that the three crystal structures of the BiVO_4_ could be mutually converted under different conditions [16]. However, after becoming irradiated by a certain energy of the photon, the photoelectron generated by the BiVO_4_ was found to have a short separation time from the photo-hole, which led to minimal photon efficiency [17]. This factor was observed to largely limit the applications and development of the BiVO_4_.

In recent years, the studies regarding BiVO_4_ have mainly included the examination of the morphology [18] and the construction of a BiVO_4_-based heterojunction catalyst, as well as improvements in the preparation methods [19]. These improved methods were mainly determined in order to increase the separation times of the photogenic electron pairs and thereby increase the photocatalytic capacity of the BiVO_4_. However, many factors are known to influence BiVO_4_. For example, different preparation methods can affect the morphology, crystal types, and other properties of BiVO_4_ [20]. Additionally, the addition of additives was found to regulate the formation of its morphology [21]. It has also been observed that different materials have serious influences on the physical and chemical properties of BiVO_4_. In addition, a new BiVO_4_ composite has been synthesized by doping metal or other semiconducting materials [22], and this has become a major research focus in the field of photocatalytic materials.

The main preparation methods of BiVO_4_ include the following: high-temperature solid phase methods; hydrothermal methods; chemical precipitation methods [23]; sol-gel methods [24]; microwave synthesis methods [25], and so on. Among these methods, hydrothermal synthesis technology has been more widely studied. In the BiVO_4_ synthesis process using hydrothermal methods, the pH value of the precursor solution, reaction temperatures, reaction times, and precursor solution concentrations are the main factors affecting the nanoparticle sizes, crystal forms, morphology, and specific surface areas. In this research study, the variable hydrothermal times during the synthesis of the BiVO_4_ were controlled in order to explore the differences in the crystal structures, morphology, specific surface areas, and photocatalytic performances of the BiVO_4_ generated under different hydrothermal times. In this paper, methylene blue was used as a probe to investigate the photocatalytic performance of BiVO_4_ toward emerging organic contaminants [26]. Additionally, the generation processes of BiVO_4_ with different crystallinity phases and their photocatalytic performances were further studied to pave the way for the low-cost strategy of enhancing the photocatalytic performance of BiVO_4_ applied in water treatment and environmental protection.

## 2. Materials and Methods

### 2.1. Experimental Materials

In this study, the Bi(NO_3_)_3_·5H_2_O (AR) was obtained from Tianjin Kemiou Chemical Reagent Co., Ltd.; the NH_4_VO_3_ (AR) was provided by the Tianjin Fuchen Chemical Reagent Factory; the NaOH (AR) was obtained from Xilong Scientific Co., Ltd.; Tianjin Fuyu Fine Chemical Co., Ltd. provided the absolute ethyl alcohol (AR); and the HNO_3_ (AR) used in this study was obtained from Guangdong Guanghua Sci-tech Co., Ltd.

### 2.2. Preparation Methods

**Figure 1:**
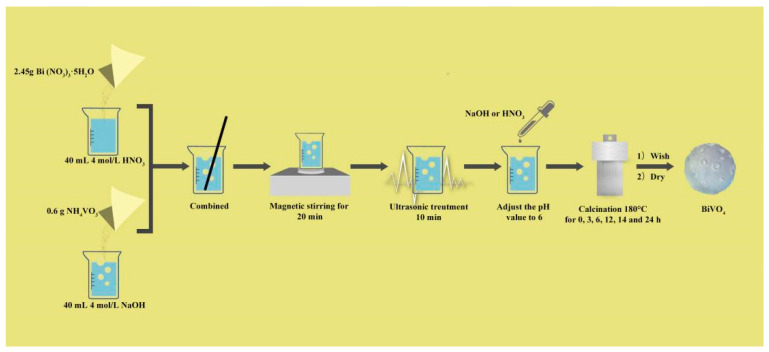
BiVO_4_ preparation flow chart.

In this research study, under the conditions of no template and no surfactant, 2.45 g of Bi(NO_3_)_3_·5H_2_O and 0.6 g of NH_4_VO_3_ were dissolved in 40 mL of 4 mol/L HNO_3_ solution and NaOH solution, respectively. Then, after Bi(NO_3_)_3_·5H_2_O and NH_4_VO_3_ were fully dissolved, the two solutions were combined, followed by magnetic stirring for 20 min and sonification for 10 min. The NaOH and HNO_3_ solution was used to adjust the pH value to 6. Then, the obtained solution was placed in a static condition overnight and transferred to a reaction kettle with a Teflon-lined stainless-steel autoclave, which was followed by a reaction at 180 °C for 0, 3, 6, 12, 14, and 24 h periods. When they had been cooled to room temperature, the as-prepared samples were washed three times with deionized water and ethanol. Then, centrifugal filtering was performed, and a six-hour drying process at 80 °C was completed in order to obtain the purified products [27]. The preparetion illustration is presented as Figure 1. The hydrothermal times were 0, 3, 6, 12, 14, and 24 h, and the prepared products were, respectively, referred to as t = 0 BiVO_4_; t = 3 BiVO_4_; t = 6 BiVO_4_; t = 12 BiVO_4_; t = 14 BiVO_4_; and t = 24 BiVO_4_. 

### 2.3. Characterizations

The BiVO_4_ structure was measured using X-ray powder diffraction (XRD, Smart Lab (3 KW)) between 10° and 75°, which used Cu K radiation (=0.15406 nm) produced at 40 kV and 40 mA. The JCPDS (Joint Committee on Powder Diffraction Standards) files were compared to identify the crystalline phases. The morphological examination used a field emission scanning electron microscope (SEM, FEI Quanta 400 FEG) with an energy dispersive X-ray analyzer system (EX-250). The specific surface area of the samples was characterized using a TriStar II 3020 surface area analyzer at 77 K. UV-Vis diffuse reflection spectra (DRS) were collected with a UV-Vis spectrophotometer (Lambda, 650).

### 2.4. Photocatalytic Activity Tests

30 mg of the BiVO_4_ prepared in the above-mentioned process was weighed and placed into 20 mg/L of methylene blue solution (30 mL), and then stirred under dark conditions for thirty minutes. The solution was then irradiated by visible light with a wavelength λ > 420 nm (550 W xenon lamp illumination, BL-GHX-V, Shanghai Bilang Instrument Manufacturing Co., Ltd.). The distance from the light source to the photocatalytic reactor was set as 10 cm. A shading system was equipped to ensure that the photocatalytic degradation was not affected by the light from outside. Sampling was conducted every 30 min, and the supernatant was collected in order to measure the absorbance after centrifugation.

## 3. Results and Discussion

### 3.1. X-ray Diffraction (XRD) 

**Figure 2:**
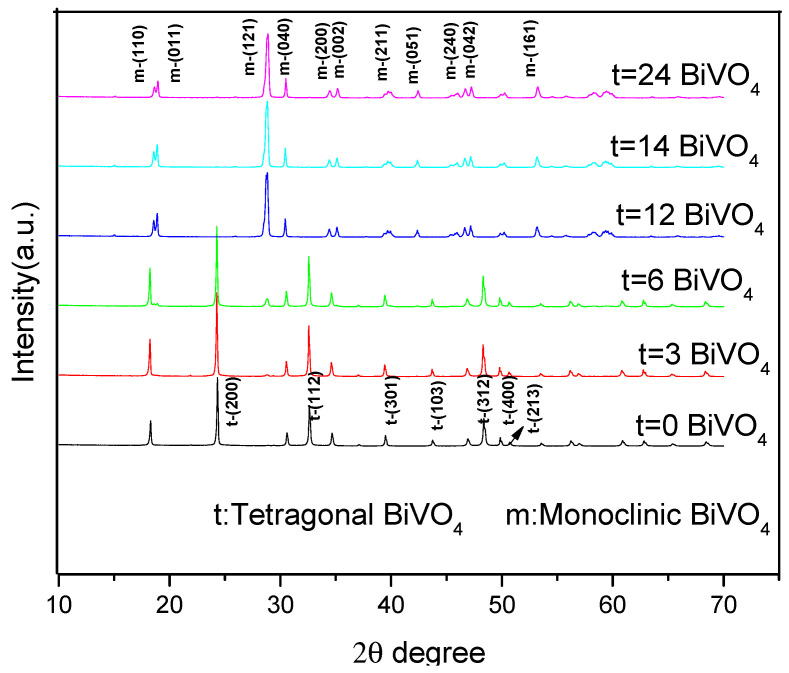
XRD spectra of the BiVO_4_ prepared under different hydrothermal time conditions.

Figure 2 details the X-ray diffraction patterns when the hydrothermal times were 0, 3, and 6 h, and 2 θ = 18.6°, 18.8°, 24.9°, 28.8°, 30.4°, 33.1°, 35.1°, 39.9°, 42.4°, 46.0°, 46.6°, 47.2°, 50.2°, 53.2°, 58.2°, and 59.4° with the diffraction peaks. When the hydrothermal times were zero and three hours, the obtained BiVO_4_ was found to display tetragonal diffraction peaks. When the hydrothermal time was six hours, a weak diffraction peak (121) in the monoclinic BiVO_4_ appeared. These findings indicated that when the hydrothermal times were zero and three hours, the tetragonal BiVO_4_ was obtained. However, when the hydrothermal time was six hours, the mixed crystals of the tetragonal and monoclinic phases were obtained. It was found that when the hydrothermal times were 12, 14, and 24 h, the characteristic peaks at 2 θ values of 18.6°, 18.8°, 28.8°, 30.4°, 35.1°, 39.9°, 42.4°, 46.0°, 46.6°, 47.2°, 50.2°, 53.2°, 58.2°, and 59.4° were consistent with the (110), (011), (120), (040), (200), (002), (211), (150), (024), (202), (161), (321), and (123) of the monoclinic BiVO_4_ (PDF NO. 75-1866). Therefore, the resulting product was determined to be a single monoclinic BiVO_4_ crystal [28]. It was also indicated that the times of the hydrothermal reactions were directly related to the crystal shape of the BiVO_4_. Furthermore, the longer hydrothermal times were found to be more conducive to the generation of the monoclinic BiVO_4_. The results of this study indicated that the generation process of the BiVO_4_ crystals was as follows: The tetragonal phase to mixed crystals of the tetragonal and monoclinic phases, and then to a single monoclinic phase. 

In order to better clarify the influence degrees of the hydrothermal times on each crystal plan growth of the BiVO_4_, (011) was taken as the internal standard of the diffraction peak to normalize the XRD diffraction peak intensity. Table 1 lists the (121)/(011) and (040)/(011) intensity of each diffraction peak. It was observed that when t = 6 h, the (121) and (040) diffraction peaks were relatively weak, and the (121)/(011) and (040)/(011) values were 0.215 and 0.408. However, with the increases in the hydrothermal times, the (121) and (040) diffraction peaks were gradually strengthened, which indicated that certain hydrothermal times were conducive to the formations of the (121) and (040) diffraction peaks. In the m-BiVO_4_ standard PDF card (JCPDS No. 140688), the (040)/(121) intensity ratio is 0.25, which has been taken as the standard. When t = 6 h, the (040)/(121) had a value of 1.897, which indicated a weak (121) diffraction peak intensity. Then, as the hydrothermal times increased, the (040)/(121) values were determined to be 0.261, 0.296, and 0.307, which were close to the (040)/(121) values of 0.25 in the m-BiVO_4_ standard PDF card (JCPDS No. 140688). In this study, when t = 12 h, the value of 0.261 was almost approached, which confirmed that that particular hydrothermal time was favorable for the formation of m-BiVO_4_ diffraction peaks. However, when t was 14 and 24 h, it was observed that the (040)/(121) values displayed slightly increasing trends, indicating that when the hydrothermal time was 12 h, the value of the (040)/(121) was the most similar to the ratio in the m-BiVO_4_ standard PDF card (JCPDS No. 140688).

### 3.2. UV-Vis Test Analysis

**Figure 3:**
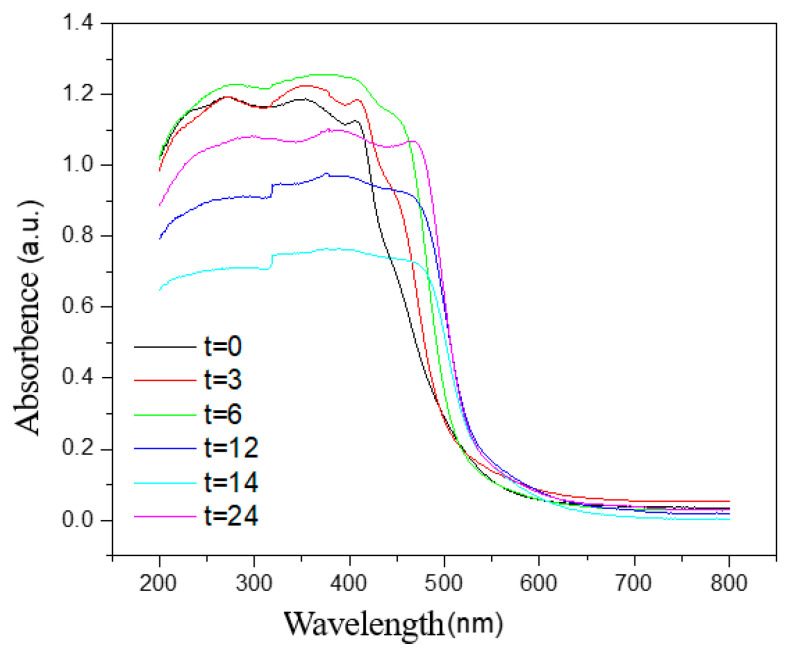
UV-Vis spectra of the BiVO_4_ under the different hydrothermal time conditions.

Figure 3 details the UV-Vis spectra of BiVO_4_ under different hydrothermal time conditions. It can be seen that the samples in the range of 520 to 580 nm displayed strong absorption abilities. Additionally, the sharp drop in the regional absorbing boundary was due to the transition of the electrons in the semiconductor material during the absorption of certain energies [12]. It can be seen from the figure that when the hydrothermal time was 12 h, 14 h, and 24 h, the absorption band was more red-shifted than at 0 h, 3 h, and 6 h. This was found to be consistent with the conclusions reached regarding the BiVO_4_ XRD spectrum. Therefore, it can be seen that during the period ranging from 0 to 12 h, with the increases in the hydrothermal times, the absorption band of the BiVO_4_ generated the redshift, and its band gap also decreased. However, when the hydrothermal times were 14 and 24 h, its redshift was not obvious, which may be related to the agglomeration of the BiVO_4_ crystals, along with the diameter sizes of the crystals.

### 3.3. SEM Test Analysis

**Figure 4:**
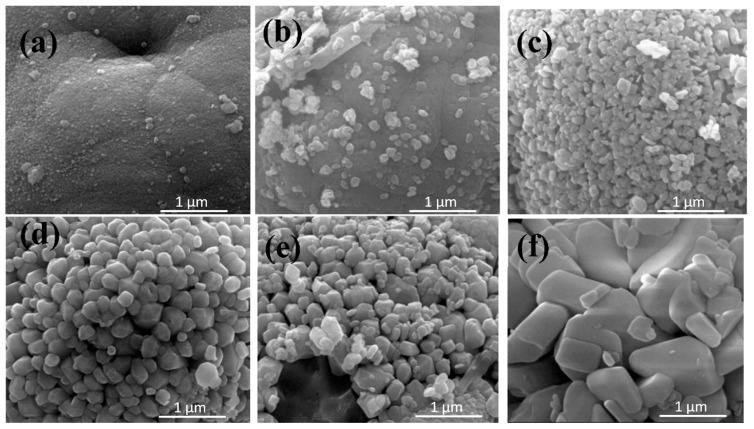
SEM maps of the BiVO_4_ prepared at different hydrothermal times with an observation diameter of 1 μm: (**a**) 0, (**b**) 3, (**c**) 6, (**d**) 12, (**e**) 14, (**f**) 24 h.

As can be seen from the SEM image displayed in Figure 4, as the hydrothermal time increased, the shape generally became spheroid. Additionally, when the hydrothermal times were 0 and 3 h, its spherical surfaces were relatively smooth. When the hydrothermal times were 6, 12, 14, and 24 h, the smooth spherical surfaces became gradually differentiated into many small polyhedron crystal pieces. Then, as the lengths of the hydrothermal times increased, the volumes of polyhedron crystals on the spherical surfaces were observed to gradually increase. Moreover, when the hydrothermal times were 14 and 24 h, the spheroids gradually showed hollow disintegration trends, and some differentiated polyhedron crystals appeared to be separated from the spheres. It was concluded that, according to the aforementioned process, the BiVO_4_ generation process could be speculated as follows: First, a smooth spheroid material was formed due to precipitation; then, the material was gradually differentiated and tiny particles were generated on the surfaces. These tiny particles gradually formed into polyhedron crystals. As the polyhedron crystals gradually increased, the agglomeration and clustering processes appeared to form chrysanthemum-like shapes (Table 2). Moreover, it was found that when the hydrothermal time was 24 h, the crystal particle sizes were significantly larger than those observed at the 14 h and 12 h stages, and serious agglomeration was evident. These findings indicated that the high-temperature and high-pressure conditions during the process of hydrothermal synthesis were favorable to the generation of monoclinic BiVO_4_ crystals. However, as the hydrothermal time increased, the agglomeration phenomenon of the BiVO_4_ crystals seemed to become more serious.

**Figure 5:**
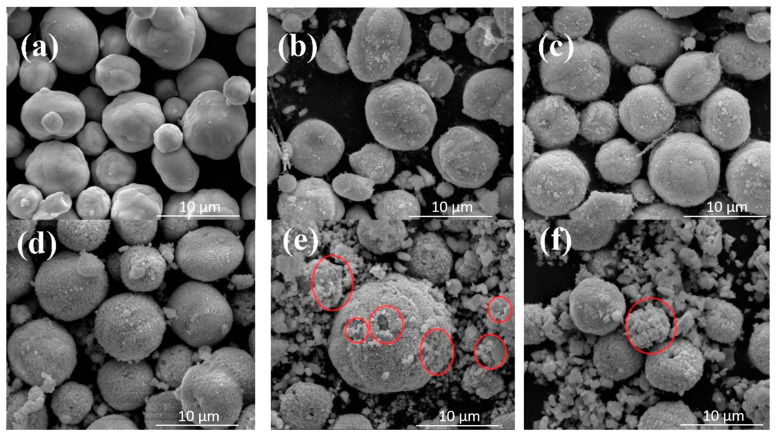
SEM maps of the BiVO_4_ prepared at different hydrothermal times with an observation diameter of 10 μm: (**a**) 0, (**b**) 3, (**c**) 6, (**d**) 12, (**e**) 14, (**f**) 24 h.

Figure 5 details this study’s SEM map with an observational diameter of 10 μm. It can be seen in the figure that when the hydrothermal times were 0, 3, and 6 h, circular spherical particles with uniform distributions and no agglomeration phenomena were evident. However, when the duration of the hydrothermal time was more than 12 h, it was observed that many small particles were present. These polyhedron crystals were the crystal particles that had been detached from the matrix. When the hydrothermal time was fourteen hours, many large concave parts were presented on the spherical surfaces (within the red circle highlighted in Figure 5). These parts were determined to be the traces of the BiVO_4_ crystals that had been detached from the matrix, which confirmed the speculation that the BiVO_4_ crystals were detached from the matrix.

The results of this study’s nitrogen adsorption experiment were carried out using a Tristar 3020 specific surface area analyzer which showed that when the hydrothermal times were 0, 3, 6, 12, 14, and 24 h, the respective specific surface areas were 2.5952 m^2^/g, 2.6115 m^2^/g, 2.7727 m^2^/g, 2.9271 m^2^/g, 3.0971 m^2^/g, and 2.0352 m^2^/g (Table 2). These results confirmed that with the increases in the durations of the hydrothermal times, the specific surface areas of the BiVO_4_ crystals displayed increasing trends. However, when the hydrothermal time was 24 h, it was determined that the causes of the sharp declines in specific surface areas were largely related to the agglomeration of the BiVO_4_ crystals [29].

### 3.4. FT-IR and Raman Analyses

**Figure 6:**
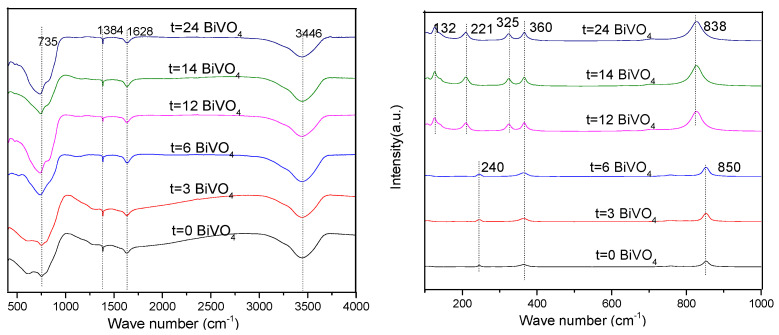
FTIR (**left**) and Raman spectra (**right**) of the BiVO_4_ prepared with different hydrothermal times.

As can be seen from the infrared spectrum detailed in Figure 6, the absorption bands in the areas of 3446 cm^−1^, 1628 cm^−1^, and 1384 cm^−1^ were different forms of vibrations generated by the H-O-H bond in the water molecules. Meanwhile, in the area of 735 cm^−1^, a visible vibration peak had occurred, which had been generated by the VO_4_^3−^ tetrahedron in the BiVO_4_ crystals. As can be seen in the figure, when t = 0, 3, and 6 h, the Raman spectra displayed the same type of Raman peak. However, when t = 12, 14, and 24 h, another similar type of Raman peak was presented. Among these, the 838 cm^−1^ and 850 cm^−1^ peaks represented the Raman peaks of the V-O bonds in the monoclinic and tetragonal BiVO_4_, respectively, while the peaks at 360 cm^−1^ and 325 cm^−1^ were the V-O symmetric bending mode (Ag) and antisymmetric bending mode (Bg) of the VO_4_^3−^ group, respectively. The peak at 221 cm^−1^ was the outer mode generated by the spin and frequency shift of the V-O bond in the VO_4_^3−^ group. These results were found to be consistent with those of the XRD analysis. The internal structures of the BiVO_4_ with different crystal types were observed to be diversified, which resulted in different Raman peaks [30].

### 3.5. BiVO_4_ Generation Mechanism

**Figure 7:**
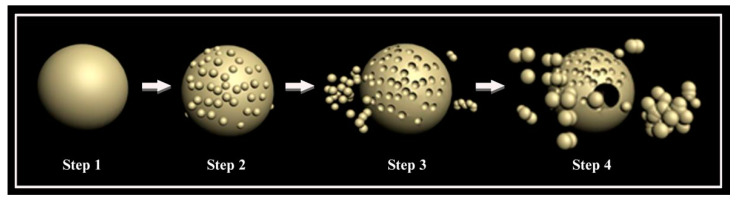
Prediction diagram of the BiVO_4_ generation mechanism.

As displayed in Figure 7, with the extension of the hydrothermal time, the morphological change of BiVO_4_ had four steps [12]:

Step 1 Oswald ripening process: During the process of the precursor solution preparation and overnight static placement, tiny particles were observed to have been deposited on the surfaces of the larger particles, which gradually formed into spherical particles with smooth surfaces [31].

Step 2 Particle differentiation process: It was observed that with the increases in the temperature and hydrothermal times, many fine crystal particles were gradually differentiated onto the smooth surfaces;

Step 3 Crystal detachment: It was found that with the further extension of the durations of the hydrothermal times, the spheroid matrix structure became damaged, and the surface differentiated crystals had gradually fallen off;

Step 4 Crystal agglomeration: It was further observed that with the continued extensions of hydrothermal times, the polyhedron crystals had gradually separated from the matrix and agglomeration occurred. Moreover, with the increases in the durations of the hydrothermal times, the BiVO_4_ crystals gradually transformed from single tetragonal and tetragonal-monoclinic mixed crystals to monoclinic crystals. These findings indicated that the tetragonal phase had converted to a monoclinic phase as the hydrothermal time increased. Meanwhile, the results of this study’s degradation experiment using methylene blue solution showed that the photocatalytic performances of the monoclinic BiVO_4_ crystals were superior to those of the tetragonal BiVO_4_ crystals. However, the agglomeration of the BiVO_4_ crystals tended to weaken their photocatalytic performances [32]. 

### 3.6. Photocatalytic Performance Analysis

**Figure 8:**
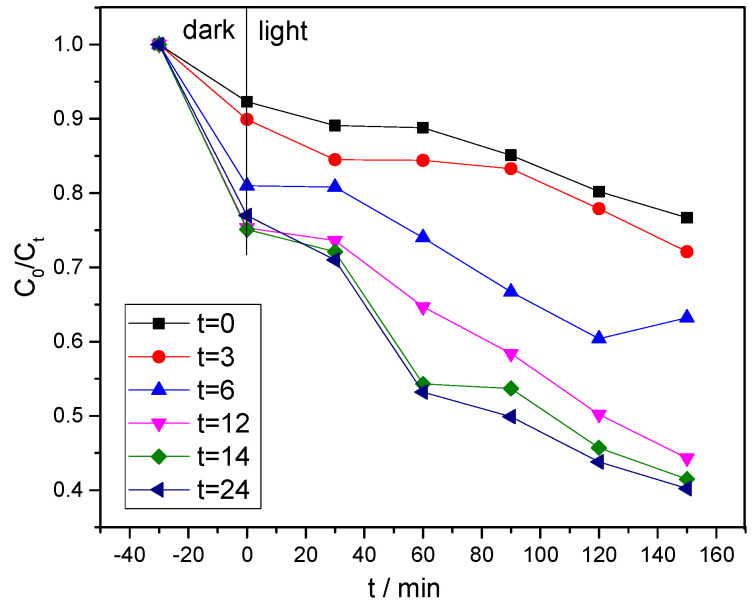
Degradation curves of the methylene blue solution under the different BiVO_4_ degradation conditions.

It can be seen in Figure 8 that, when the hydrothermal time was 24 h, the BiVO_4_ displayed the best activity. Meanwhile, when hydrothermal times were zero and three hours, the degradation performances of the BiVO_4_ were observed to be somewhat less effective than when the hydrothermal times were 6, 12, 14, and 24 h. These results were found to be consistent with the absorption boundary values reflected by the solid ultraviolet. It was found that as the durations of the hydrothermal times increased, the photocatalytic performances of the prepared products, as well as the adsorption capacity of the methylene blue solution, were gradually and successively increased. However, when t = 24 h, the adsorption ability of the BiVO_4_ was found to be slightly weaker than when t = 14 h. This was determined to be related to the BiVO_4_ surface area. The degradation curve of the MB further confirmed that the photocatalytic performances of the monoclinic BiVO_4_ were superior to those of the tetragonal BiVO_4_ [25], which is consistent with the references that monoclinic BiVO_4_ possessed superior separation of the photo-induced charge carriers to that of tetragonal BiVO_4_ [12]. Additionally, the photocatalytic activity of BiVO_4_ in our study was compared with other references and is presented in Table 3. Furthermore, it was reported that the heterojunction structure between tetragonal BiVO_4_ and monoclinic BiVO_4_ could exhibit much higher photocatalytic activity [27], which is worth further exploration. 

## 4. Conclusions

In this work, BiVO_4_ was successfully constructed through the hydrothermal method under different hydrothermal times. XRD results showed that the longer hydrothermal times were found to be more conducive to the generation of the monoclinic BiVO_4_. The UV-Vis absorption was more red-shifted when hydrothermal time increased from 3 h to 24 h. SEM results showed that the high temperature and high pressure conditions in the hydrothermal synthesis process are conducive to the formation of monoclinic BiVO_4_ crystals, but the agglomeration phenomenon of BiVO_4_ becomes more serious with the increase in hydrothermal time. The specific surface area of all the samples was in the range of 2.0~3.1 m^2^/g. Moreover, BiVO_4_ had excellent photocatalytic performance toward MB. Additionally, the photocatalytic activity shows that the crystal phase of BiVO_4_ plays a vital role in the photocatalytic performances: the removal efficiency of monoclinic BiVO_4_ (45% in 150 min), including adsorption and photocatalysis, was more than twice that of the tetragonal BiVO_4_ (20% in 150 min), which provides a facile guideline for the photocatalytic degradation of emerging organic contaminants using BiVO_4_. 

## Figures and Tables

**Table 1:** XRD microstructure parameters of the BiVO_4_ products prepared under different hydrothermal conditions.

Sample	Time	Crystal Structure	Diffraction Intensity/CPS				Relative Intensity Ratio		
			m-(121)	m-(040)	t-(200)	t-(112)	(121)/011	(040)/(011)	(040)/(121)
BiVO_4_	0 h	t + m	--	5909	29,744	17,675	--	0.537	--
	3 h	t + m	--	6663	36,229	21,987	--	0.410	--
	6 h	t + m	3610	6848	34,808	21,794	0.215	0.408	1.897
	12 h	m	27,973	7297	--	--	3.758	0.942	0.261
	14 h	m	28,627	8476	--	--	2.884	0.854	0.296
	24 h	m	27,740	8516	--	--	3.754	1.152	0.307

**Table 2:** Related physical properties of the BiVO_4_ crystals.

Hydrothermal Time/h	Crystal Phase	Morphology	Specific Surface Areas m^2^/g	Reasons for Changes in Specific Surface Area
0	Tetragonal	Smooth spheroid	2.5952 ± 0.1498	Surface differentiation
3	Tetragonal	Relatively smooth spheroid	2.6115 ± 0.1642	
6	Tetragonal + minor monoclinic	Surface-differentiated polyhedron	2.7727 ± 0.1581	
12	Monoclinic	Clustered flower shape	2.9271 ± 0.1085	
14	Monoclinic	Clustered flower shape	3.0971 ± 0.1525	
24	Monoclinic	Polyhedron agglomeration	2.0352 ± 0.1276	Crystal agglomeration

**Table 3:** Photocatalytic activity of BiVO_4_ toward different dyes under visible light irradiation.

Materials	Photocatalytic Parameters	Degradation Efficiency	Refs.
BiVO_4_ peanut, 1.0 mg/mL	MB, 10 mg/L, Xenon lamp with 35 W/m^2^	40% in 120 min	[33]
BiVO_4_ microtube, 1.0 mg/mL	MO, 20 mg/L, 250-W Xenon arc lamp	95% in 180 min	[34]
BiVO_4_ spheres, 0.5 mg/mL	RhB, 10^−5^ mol/L, 500 W Xenon lamp	27% in 150 min	[35]
BiVO_4_ biscuits, 0.5 mg/mL	RhB, 10^−5^ mol/L, 500 W Xenon lamp	44% in 150 min	[35]
Tetragonal BiVO_4_, 1.0 mg/mL	MB, 20 mg/L, 550 W Xenon lamp	20% in 150 min	This study
Monoclinic BiVO_4_, 1.0 mg/mL	MB, 20 mg/L, 550 W Xenon lamp	45% in 150 min	This study

## Data Availability

Data available on request from the authors.
